# Pure erythroid leukemia subsequent to acute myelomonocytic leukemia: A case report

**DOI:** 10.1097/MD.0000000000025528

**Published:** 2021-04-16

**Authors:** Jiamei Ji, Yating Li, Lei Fan, Hua Lu, Xiaoyan Qu

**Affiliations:** a Department of Hematology, The First Affiliated Hospital of Nanjing Medical University, Key Laboratory of Hematology of Nanjing Medical University; b Collaborative Innovation Center for Cancer Personalized Medicine, Nanjing, China.

**Keywords:** acute myelomonocytic leukemia, diagnosis, prognosis, pure erythroid leukemia

## Abstract

**Rationale:**

Pure erythroid leukemia is a rare subcategory of acute myeloid leukemia characterized by predominant immature erythroid population. Its occurrence subsequent to acute myelomonocytic leukemia has not been reported before. We reported this rare case to call attention because it may pose a diagnostic challenge.

**Patient's concerns:**

A 54-year-old female patient presented to our hospital in March 2018 with symptoms of easy fatigability.

**Diagnosis:**

Bone marrow aspiration was hypercellular showing 67.2% blasts mainly including moderate myeloblasts and monoblasts. There was mild dysplasia with some cells having round, oval, or bizarre nuclei which containing 1 to 3 nucleolus. Erythroid lineage was hypoplasia and mature erythrocytes were generally normal. Conventional cytogenetics of bone marrow cells revealed complex karyotype (44, XX, del (5) (q14q34) del (5) (q14q34), del (14) t (11;14) (q10; q10), −16, del (17), −18[10]).

**Interventions:**

The patient was treated with second line chemotherapy but did not respond.

**Qutcomes:**

She died of cardiopulmonary failure 19days after starting of therapy.

**Lessons:**

This unexpected and relatively uncommon occurrence was associated with a universally rapid and fatal clinical course with survival measured in <2 months despite intensive chemotherapy. We call attention to this rare phenomenon because it may pose a diagnostic challenge.

## 1 Introduction

Pure erythroid leukemia (PEL) is an extremely uncommon subcategory of acute myeloid leukemia (AML). This disease was defined as a neoplastic proliferation of either proerythroblasts or undifferentiated immature cells committed exclusively to the erythroid lineage comprising >80% of all bone marrow nucleated elements and no significant myeloblast population by the World Health Organization (WHO).^[1]^

A significant fraction of patients with AML have a prior history of an antecedent malignancy (hematologic or otherwise) treated with chemotherapy and/or radiotherapy.^[2]^ Therapy-related AML (t-AML) included in the therapy-related myeloid neoplasms category of the 2016 WHO classification,^[1]^ as a second primary malignancy (SPM) is usually a well-known late effect after cancer. The occurrence of another subtype of AML after AML treatment was extremely rare. Herein, we reported one case, who presented with PEL only 4 months after diagnosis of acute myelomonocytic leukemia.

## 2 Case report

A 54-year-old female patient presented to our hospital in March 2018 with symptoms of easy fatigability. She had hemoglobin (Hb) of 50 g/L, white blood cell (WBC) 18.79 × 10^9^/L and reduced platelets at 44 × 10^9^/L. Physical examination of the patient showed pallor and splenomegaly of 2 cm below left costal margin with rest of examination unremarkable.

Investigations revealed as under: Hb 52 g/L, WBC 22.12 × 10^9^/L, neutrophil 32.7%, lymphocyte14.8%, eosinophil 1.9%, basophil 1.2%, platelets 57 × 10^9^/L, Retics 0.97%. Lactate dehydrogenase (LDH) 1189 U/L (normal ranges: 140–271U/L). Iron kinetics showed ferritin 524.1 (normal ranges: 23.9–336.2) ng/mL. Coagulogram was within normal limits.

**Figure 1:**
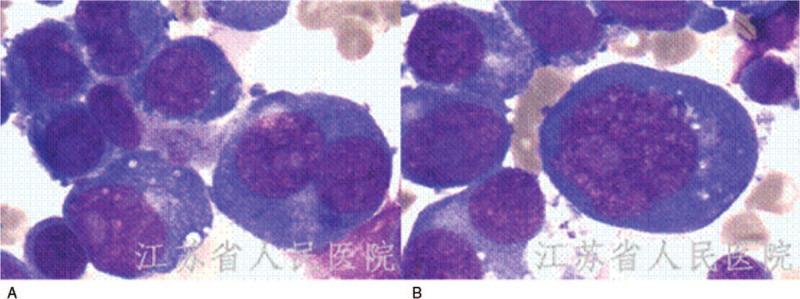
Bone marrow aspirate with moderate myeloblasts and monoblasts at initial diagnosis of acute myelomono cytic leukemia (A). Bone marrow smears showing several erythroblasts at diagnosis of pure erythroid leukemia(B).

Bone marrow aspiration (Fig. 1A) was hypercellular showing 67.2% blasts mainly including moderate myeloblasts and monoblasts. There was mild dysplasia with some cells having round, oval or bizarre nuclei which containing 1 to 3 nucleolus. Erythroid lineage was hypoplasia and mature erythrocytes were generally normal. Myeloperoxidase (MPO), diffuse Periodic acid-Schiff (PAS) and non-specific esterase (NSE) positive erythroblasts were seen and constituted 72%, 34%, and 41% of total cells. Flow cytometry immunophenotyping of the bone marrow aspirate exhibited bright expression of CD34, CD117, HLA-DR, CD64, CD14, CD4, CD15, CD56, CD33, CD13, CD38, CD58, CD123, CD81, CD11b on blasts. The blasts were negative for CD19, CD22, CD20, CD10, CD2, CD7, CD5, CD3, CD8. The next generation sequencing (NGS) showed TET2, GATA2 SNP, and TP53 mutation, along with TP53 deletion. Conventional cytogenetics of bone marrow cells revealed complex karyotype (44, XX, del (5) (q14q34) del (5) (q14q34), del (14) t (11;14) (q10; q10), −16, del (17), −18[10]). Diagnosis of acute myelomonocytic leukemia was made.

AML chemotherapy was started with IA regime (idarubicin 10 mg/m^2^/day, days 1–3; cytarabine 100 mg/m^2^/day, days 1–7). The patient did not respond to the induction chemotherapy, with the blasts from 67.2% to 52.4% after one cycle treatment. The immunophenotype of blasts was consistent with initial diagnosis. Considering she had TET2 mutation, we performed salvage chemotherapy consisting of decitabine (15 mg/m^2^/day on days 1–5) plus FLAG (fludarabine 30 mg/m^2^/day days 1–5, cytarabine 2 g/m^2^/day days 1–5, and 300 μg/day G-CSF from day 0) regimen. The patient went into complete remission after salvage chemotherapy. She then received consolidation chemotherapy of one more cycle of decitabine plus FLAG. However, her Hb, WBC and platelets count still did not recover 1 month after consolidation therapy (Hb 70 g/L, WBC 1.1 × 10^9^/L, and platelets 57 × 10^9^/L). The patient's condition deteriorated quickly along with persistent high fever. Biochemical markers revealed elevated serum levels of LDH 2067U/L.

Bone marrow aspiration (Fig. 1B) yielded a markedly hypercellular bone marrow essentially replaced by erythroid precursors, representing approximately 86% of the marrow cells. Blasts were predominantly medium to large with high nuclear to cytoplasmic ratio, round nuclei, fine reticular chromatin, prominent nucleoli. No significant dysplastic changes were identified in non-erythroid lineages MPO was negative in these cells. PAS stain showed cytoplasmic block positivity in the erythroid precursors. On immunophenotypic analysis by CD45 negative/low side scatter gating, there were two populations of cells. One population consisted of 4.3% expressed CD34, CD117, CD33, CD13, HLA-DR, CD58, CD81, CD123, and CD56 and were CD10, CD7, CD4, CD15, CD19, CD64, CD14 negative. The other immature cell population consisted of 36.9% which were positive for CD71, glycophorin A, CD58, CD81, and partially positive for CD117, consistent with erythroid precursors. Staining for CD13, CD33, CD34, and all other myeloid or lymphoid makers was negative. The NGS showed TET2, TP53, KRAS, and GATA2 SNP mutation, along with TP53 deletion. The overall clinical, morphological, cytochemical and immunophenotypic findings suggested a diagnosis of PEL. The patient was treated with salvage chemotherapy regimen consisting 5 mg/m^2^/day cladribine (days 1–5); 200 mg/m^2^/day cytarabine (days 1–7), and 300 μg/day G-CSF (days 0–5), but the patient started to have left upper quadrant pain with fever from the first day of chemotherapy. The chest and abdominal CT scan with contrast demonstrated pulmonary infection and splenic infarction. The patients started to have frequent arrhythmias such as atrial flutter and atrial fibrillation. Unfortunately, after starting of therapy, the patient died 19 days later of heart failure and pneumonia. Bone marrow at 15 days after treatment showed no response.

## 3 Discussion

Acute erythroleukemia (according to WHO 2008 Criteria) constitutes about 1% of overall de novo AML and especially occurs in older patients. PEL is even more rare, accounting for 3% of acute erythroid leukemia.^[3]^ Due to its rarity, studies of PEL have been limited to small series and case reports. In this report we document a rare case of adult PEL after the diagnosis of AML. The unique circumstances of our cases present significant diagnostic challenges. The diagnosis of PEL is inherently challenging because they may be morphologically similar to other leukemias and non-hematopoietic tumors,^[4]^ and in our case, PEL after AML makes the diagnosis even more difficult.

In addition to its distinct morphology, immunophenotypic analyses are necessary for PEL diagnosis. In PEL, CD34, and HLA-DR are uniquely negative in blasts. CD117 is often partially or weakly positive and some may have aberrant expression of CD33.^[5]^ Other myeloid markers as well as T-cell and B-cell markers are usually negative. Two iron-related proteins, CD71 and ferritin H, have recently emerged as sensitive and specific markers for erythroid lineage cells.^[6]^ Glycophorin A positivity has been reported to be seen in erythroid precursors.^[7]^ E-cadherin is another highly specific and sensitive marker for immature erythroblasts, but it is not lineage-specific and is positive in many other types of cells.^[8,9]^ In some cases of erythroid leukemia that glyco-phorin A is negative, staining for E-cadherin can be very important in confirming the erythroid lineage.^[10]^ As was seen in our case, the immature celles expressed CD71, glycophorin A, CD58, CD81, and partially positive for CD117, which were significantly different with first clone expressions.

At a cytogenetic level, most cases of PEL presented with a complex karyotype, median number of cytogenetic abnormalities could be 20 in previous report.^[11]^ No specific genetic mutations have been described in PEL. The most frequently mutated gene was TP53.^[6,11]^ Importantly, co-occurrence of TP53 mutations and TP53 deletions resulting from chr17 abnormalities was detected in PEL patients, suggesting more than a single TP53 abnormality may represent a pathognomonic signal associated with PEL.^[11]^ Conversely, mutations commonly found in other types of AML, such as FLT3, NPM1, and CEBPA, are very rare in PEL.^[6]^ In this patient, TP53, GATA2, TET2 mutations, TP53 deletion were identified at initial diagnosis. KRAS mutation was detected when evolution to PEL. KRAS mutation is generally associated with clinical aggressiveness of cancer and reduced survival of the patients, especially in somatic neoplasm, and in 5% of adult AML. In this case, KRAS mutation may function as cooperating mutations that was associated with disease progression and second clone development.

Distinct morphology, immunophenotyping and genetic studies help us exclude other diagnostic possibilities and make the diagnosis of PEL.

The prognosis of PEL was dismal, with a median overall survival of 1.4 to 6.6 months.^[11–13]^ The median overall survival of secondary PEL was quite similar to de novo PEL.^[11]^ The survival of our case was also poor, she did not respond to further chemotherapy after diagnosis of PEL. The OS time was only 1 month from diagnosis of PEL, and 5 months after initial diagnosis of AML. Studies have shown that there is no significant difference in OS between patients receiving induction chemotherapy and those receiving supportive therapy or hypomethylated drugs.^[14]^

Nearly half of the PEL patients in the literature were therapy-related, hydroxyurea and azathioprine accumulation have been reported to be considered to be associated with the patients’ leukemogenesis.^[15,16]^ In most cases, therapy-related AML are related to solid cancer and lymphoproliferative disease treatment. PEL as a consequence of chemoradiotherapy in the setting of myelodysplastic syndrome (MDS),^[15]^ breast cancer, chronic myeloid neoplasm, chronic lymphocytic leukemia, acute lymphoblastic leukemia, multiple myeloma has been previously reported. To the best of our knowledge, PEL after AML has not been previously reported.

The median latency interval from therapy of the primary malignancy and the diagnosis of therapy-related MDS (t-MDS) or t-AML has been reported to be 64 months for patients with an antecedent hematologic malignancy and 55 months for those with primary solid tumors.^[17]^ In this patient, the interval between the leukemias was only 4 months, thus subsequent PEL might not be considered therapy-related. This patient developed two subtypes of AML simultaneously, which was extremely rare.

Up-to-date, PEL patients have been reported to progress rapidly with poor prognosis. There is also a lack of standard treatment methods in clinical practice, which brings some challenges to the therapy. Unlike previously reported PEL cases, several noteworthy and unique findings were associated with our study. Our study is the first to describe two subtypes AML existing concurrently in one patient. This suggests that when AML patients with a highly complex karyotype, such as TP53 mutations, do not respond well to treatment, immunophenotyping is necessary to rule out the possibility of PEL. Patients developed subsequent AML are usually associated with neoplasm therapy-related or occurring secondary to an antecedent myeloid disease. This unexpected and relatively uncommon occurrence was associated with a universally rapid and fatal clinical course with survival measured in less than two months despite intensive chemotherapy. We call attention to this rare phenomenon because it may pose a diagnostic challenge.

In conclusion, we present a unique case of two subtypes AML existing in one patient. Together with previously reported treatment-related PEL cases, we believe that these cases will add to our understanding of the pathogenesis of PEL and merit further study through a multi-institutional database.

## Acknowledgments

This study was supported by National Natural Science Foundation of China (81302040).

## Author contributions

**Conceptualization:** Jiamei Ji, Xiaoyan Qu.

**Data curation:** Jiamei Ji, Yating Li, Lei Fan, Hua Lu, Xiaoyan Qu.

**Formal analysis:** Yating Li.

**Investigation:** Xiaoyan Qu.

**Resources:** Lei Fan, Hua Lu, Xiaoyan Qu.

**Writing – original draft:** Jiamei Ji, Yating Li.

**Writing – review & editing:** Xiaoyan Qu.
